# Pathological evaluation of rats carrying in-frame mutations in the dystrophin gene: a new model of Becker muscular dystrophy

**DOI:** 10.1242/dmm.044701

**Published:** 2020-09-28

**Authors:** Naomi Teramoto, Hidetoshi Sugihara, Keitaro Yamanouchi, Katsuyuki Nakamura, Koichi Kimura, Tomoko Okano, Takanori Shiga, Taku Shirakawa, Masafumi Matsuo, Tetsuya Nagata, Masao Daimon, Takashi Matsuwaki, Masugi Nishihara

**Affiliations:** 1 Department of Veterinary Physiology, Graduate School of Agricultural and Life Sciences, The University of Tokyo, Bunkyo-ku, Tokyo, 113-8657, Japan; 2 Department of General Medicine, The Institute of Medical Science, The University of Tokyo, Minato-ku, Tokyo, 108-8639, Japan; 3 Department of Cardiovascular Medicine, Graduate School of Medicine, The University of Tokyo, Bunkyo-ku, Tokyo, 113-0033, Japan; 4 Department of Laboratory Medicine, The University of Tokyo Hospital, The University of Tokyo, Bunkyo-ku, Tokyo, 113-8655, Japan; 5 Department of Veterinary Pathology, Graduate School of Agricultural and Life Sciences, The University of Tokyo, Bunkyo-ku, Tokyo, 113-8657, Japan; 6 Research Center for Locomotion Biology, Kobe Gakuin University, Nishi, Kobe, 651-2180, Japan; 7 KNC Department of Nucleic Acid Drug Discovery, Faculty of Rehabilitation, Kobe Gakuin University, Nishi, Kobe, 651-2180, Japan; 8 Department of Neurology and Neurological Science, Tokyo Medical and Dental University, Bunkyo-ku, Tokyo 113-8510, Japan

**Keywords:** Becker muscular dystrophy, Dystrophin, Rat model

## Abstract

Dystrophin, encoded by the *DMD* gene on the X chromosome, stabilizes the sarcolemma by linking the actin cytoskeleton with the dystrophin-glycoprotein complex (DGC). In-frame mutations in *DMD* cause a milder form of X-linked muscular dystrophy, called Becker muscular dystrophy (BMD), characterized by the reduced expression of truncated dystrophin. So far, no animal model with in-frame mutations in *Dmd* has been established. As a result, the effect of in-frame mutations on the dystrophin expression profile and disease progression of BMD remains unclear. In this study, we established a novel rat model carrying in-frame *Dmd* gene mutations (IF rats) and evaluated the pathology. We found that IF rats exhibited reduced expression of truncated dystrophin in a proteasome-independent manner. This abnormal dystrophin expression caused dystrophic changes in muscle tissues but did not lead to functional deficiency. We also found that the expression of additional dystrophin named dpX, which forms the DGC in the sarcolemma, was associated with the appearance of truncated dystrophin. In conclusion, the outcomes of this study contribute to the further understanding of BMD pathology and help elucidate the efficiency of dystrophin recovery treatments in Duchenne muscular dystrophy, a more severe form of X-linked muscular dystrophy.

## Abstract (precis)

**Editor's choice:** The newly established rat model carrying in-frame mutations in the *Dmd* gene exhibits the dystrophic phenotype and abnormal dystrophin expression profile, similar to patients with Becker muscular dystrophy.

## INTRODUCTION

Dystrophin (dp427), a 427 kDa protein crucial for sarcolemma integrity, is encoded by the dystrophin gene (*DMD*) on the X chromosome (Hoffman et al., 1987). Dystrophin functions as a sarcolemma-supporting protein during muscle contraction by linking the intracellular actin cytoskeleton to transmembrane components of the dystrophin-associated glycoprotein complex (DGC). The DGC is composed of several proteins represented by sarcoglycans (αSG), dystroglycans and neuronal nitric oxide synthase (nNOS) (Lapidos et al., 2004). The function of dystrophin is dependent on its N-terminal and C-terminal sequences, which are essential for binding with the cytoskeletal actin filament and with β-dystroglycan (βDG), respectively, in DGC (Campbell and Kahl, 1989; Rybakova et al., 1996). The central part of dystrophin is called the central rod domain, and is composed of 24 spectrin-like repeats (Koenig et al., 1988).

X-linked muscular dystrophy is a recessive genetic disease characterized by progressive muscle wasting. Becker muscular dystrophy (BMD) is a form of X-linked muscular dystrophy caused by in-frame mutations in *DMD.* In BMD muscle tissues, a truncated form of dystrophin (tdp427) is expressed, based on the missing genomic sequence. The pathology of this disease is generally milder than that of Duchenne muscular dystrophy, known as a more severe form of X-linked muscular dystrophy caused by the complete loss of the dystrophin protein. Patients with Duchenne muscular dystrophy show debilitating clinical symptoms, such as a difficulty in walking and standing, heart failure, and respiratory difficulties. In contrast, the clinical severity of BMD varies among individuals, from asymptomatic to as severe as Duchenne muscular dystrophy (Koenig et al., 1989).

Exon skipping is the latest therapeutic strategy for the conversion of Duchenne muscular dystrophy to BMD by skipping the mutated exons using antisense oligonucleotide (AON). Recently, the tenth AON drug was approved for use in clinical settings (Heo, 2020). In the near future, the pathology of many patients with Duchenne muscular dystrophy is expected to become as mild as that of patients with BMD, owing to such therapeutic options. However, even the latest therapy can only alleviate the clinical symptoms by converting Duchenne muscular dystrophy to BMD, but a complete cure of Duchenne muscular dystrophy and BMD still warrants future research to analyze the disease progression.

In patients with BMD, expression levels of tdp427 and DGC components are lower and more varied than those in healthy controls, and the levels are variable within single myofibers, between adjacent myofibers and between muscles (Beggs et al., 1991). Immunohistochemically, BMD muscle tissues show ‘faint and patchy’ staining of dystrophin. There is a still conflict about the use of dystrophin as a surrogate biomarker: Anthony et al. (2011) reported that the expression level of tdp427 in muscle tissues is well-correlated with the clinical severity of BMD, whereas Van Den Bergen et al. (2014) reported that tdp427 levels appear not to be a major determinant of disease severity in BMD, as long as it is above ∼10%. Although it is still under debate, a more than 90% decrease of normal dystrophin levels might have a critical effect on the clinical severity of patients with BMD. Previous studies examined the mechanisms underlying the decrease of tdp427, using *in vitro* analyses or transgenic mice that overexpress tdp427, and showed that the decreased level of tdp427 was caused by protein degradation following the tertiary instability of the protein (Henderson et al., 2011; McCourt et al., 2015, 2018). However, as patients with BMD exhibit endogenous tdp427 expression under normal promoter activity, such an *in vitro* or overexpressed tdp427 scenario might not mimic the physiological environment in muscle tissues of patients with BMD. Nevertheless, so far, no animal models carrying the in-frame mutation in the *Dmd* gene, and exhibiting reduced endogenous expression of tdp427, have been reported.

Previously, our group generated dystrophin-mutated rat strains using CRISPR/Cas-mediated gene editing targeting the 5′ region of the *Dmd* gene (Nakamura et al., 2014). Several rat strains completely lacked dystrophin protein owing to the out-of-frame mutation in *Dmd,* and have shown progressive pathology, similar to human patients with Duchenne muscular dystrophy, in their skeletal and cardiac muscles, unlike the widely used dystrophin-deficient mouse strain (mdx mice). We also generated a rat strain that carried the in-frame mutation in the 5′ region of *Dmd*. As far as we know, rats carrying an in-frame mutation in *Dmd* have not been reported, and thus, would be expected to mirror the human BMD pathology better than the previously described mouse model (McCourt et al., 2018). Moreover, the 5′ region of the *Dmd* gene (exons 2 to 20) is one of the mutational hot spots, responsible for Duchenne muscular dystrophy or BMD, that account for ∼15% of all exon deletions and ∼64% of all exon duplications within the *Dmd* gene (Tuffery-Giraud et al., 2009). Thus, this model is expected to mirror not only the human BMD pathology but also the pathology of patients with Duchenne muscular dystrophy converted to BMD by exon-skipping treatment.

In this study, we analyzed the muscular pathology in a newly established rat strain carrying in-frame mutations in the *Dmd* gene. Furthermore, we examined whether this mutation causes the pathology observed in human patients with BMD.

## RESULTS

### Establishment of a rat strain carrying an in-frame mutation in the 5′ region of *Dmd*

**Fig. 1.**
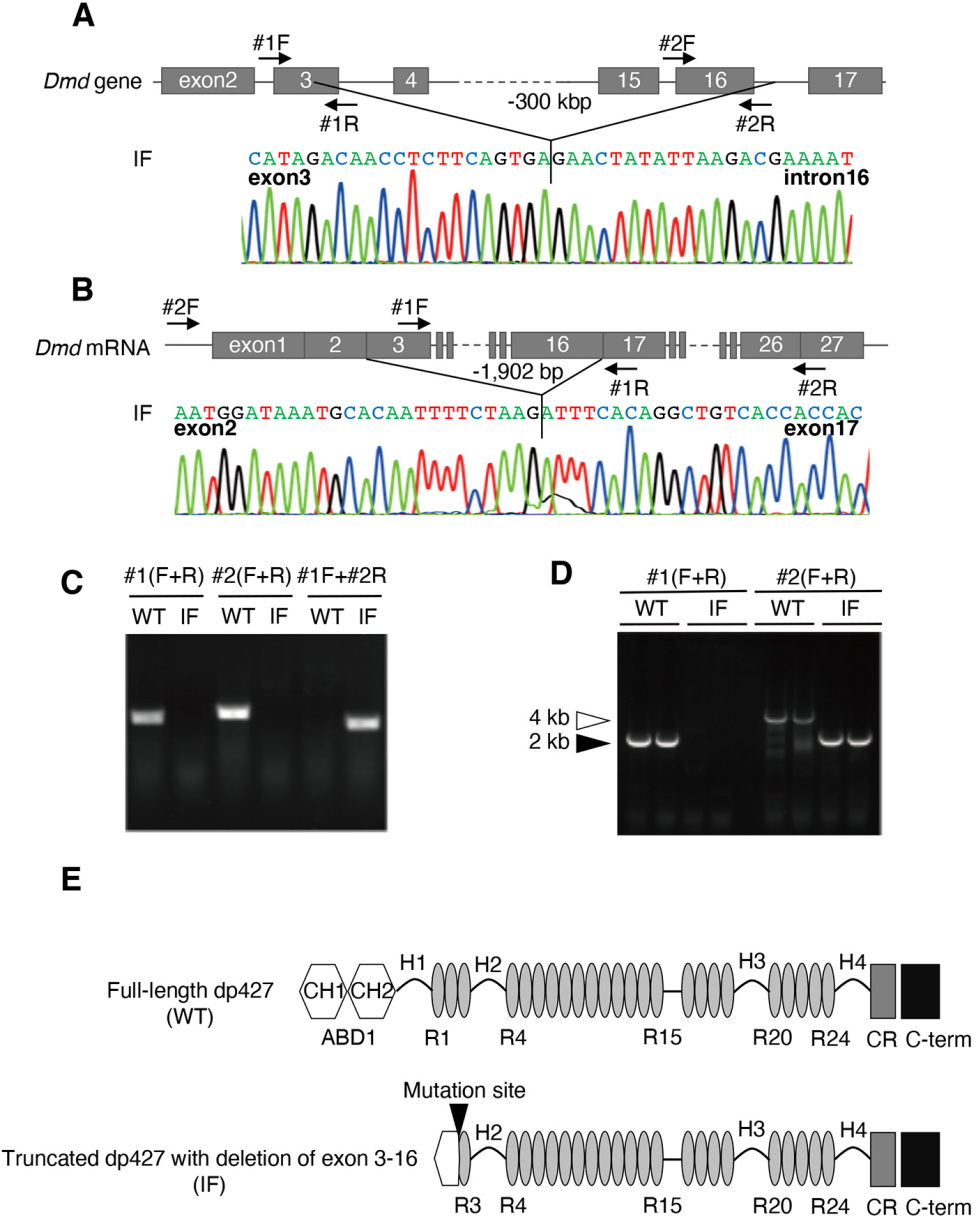
**Overview of *Dmd* gene mutations in IF rats.** (A) Sequence of the *Dmd* gene of IF rats. Arrows indicate the location of primers used in PCR. (B) Sequence of mutated *Dmd* mRNA in IF rats. Arrows indicate the location of primers used. (C) Genomic PCR results for the *Dmd* gene in wild-type (WT) and IF rats. Note the absence of PCR product in IF when primers #1R or #2F, the corresponding sequences of which are lacking in the *Dmd* gene, were used. Also note the absence of PCR product in wild type when the primer set #1F and #2R was used owing to the region being too large to amplify. (D) RT-PCR results for *Dmd* cDNA in wild-type and IF rats. Note the absence of PCR product in IF when primer #1F, the corresponding sequence of which is lacking in *Dmd* mRNA, was used. (E) The molecular structure of full-length dp427 (upper) and truncated dp427 (below). ABD1, actin-binding domain 1; CH, calponin homology domain; CR, Cysteine-rich domain; C-term, C-terminal domain; H, Hinge; R, spectrin-like repeat. The black arrowhead indicates the mutation site.

As we reported previously, mutations were introduced in rat *Dmd* using the CRISPR/Cas9 system in ten F0 dystrophin-mutated male rats (Nakamura et al., 2014). Simultaneously, a F0 *Dmd*-mutated female rat carrying a long deletion in exons 3 to 16 was acquired. In this study, we used a rat strain carrying this deletion established by crossing the F0 female rat with the wild-type Wistar–Imamichi rats. First, we checked the *Dmd* and mRNA sequence in this rat strain. Sequencing analysis and genomic PCR revealed that they inherited the deletion ranging from exon 3 to exon 16 (324,981 bp) in rat *Dmd* genome sequence (Fig. 1A,C). Next, sequencing analysis and RT-PCR showed that *Dmd* mRNA expression lacked the sequence from exon 3 to exon 16 (1902 bp), caused by the splicing switch sites (Fig. 1B,D). As the presence of in-frame *Dmd* mRNA was confirmed, this strain was designated as the IF (in-frame) strain. Exons 3 to 16 of *Dmd* translated into the part of calphonin homology domain 1 (CH1) and the entire CH2 domain, which are subdomains of N-terminal actin-binding domain 1, and the first two and part of the third spectrin-like repeats. The predicted molecular structure of tdp427 is shown in Fig. 1E.

### IF rats show typical dystrophic histopathology in skeletal muscles but not obvious functional deficiency

In patients with Duchenne muscular dystrophy and BMD, the appearance of tissue dystrophic phenotypes, such as continuous muscle degeneration and enhancement of intramuscular fibrosis/adipogenesis, result in muscle tissue dysfunction. To study whether IF rats exhibit dystrophic phenotypes in skeletal muscles, pathological evaluations were conducted.

**Fig. 2.**
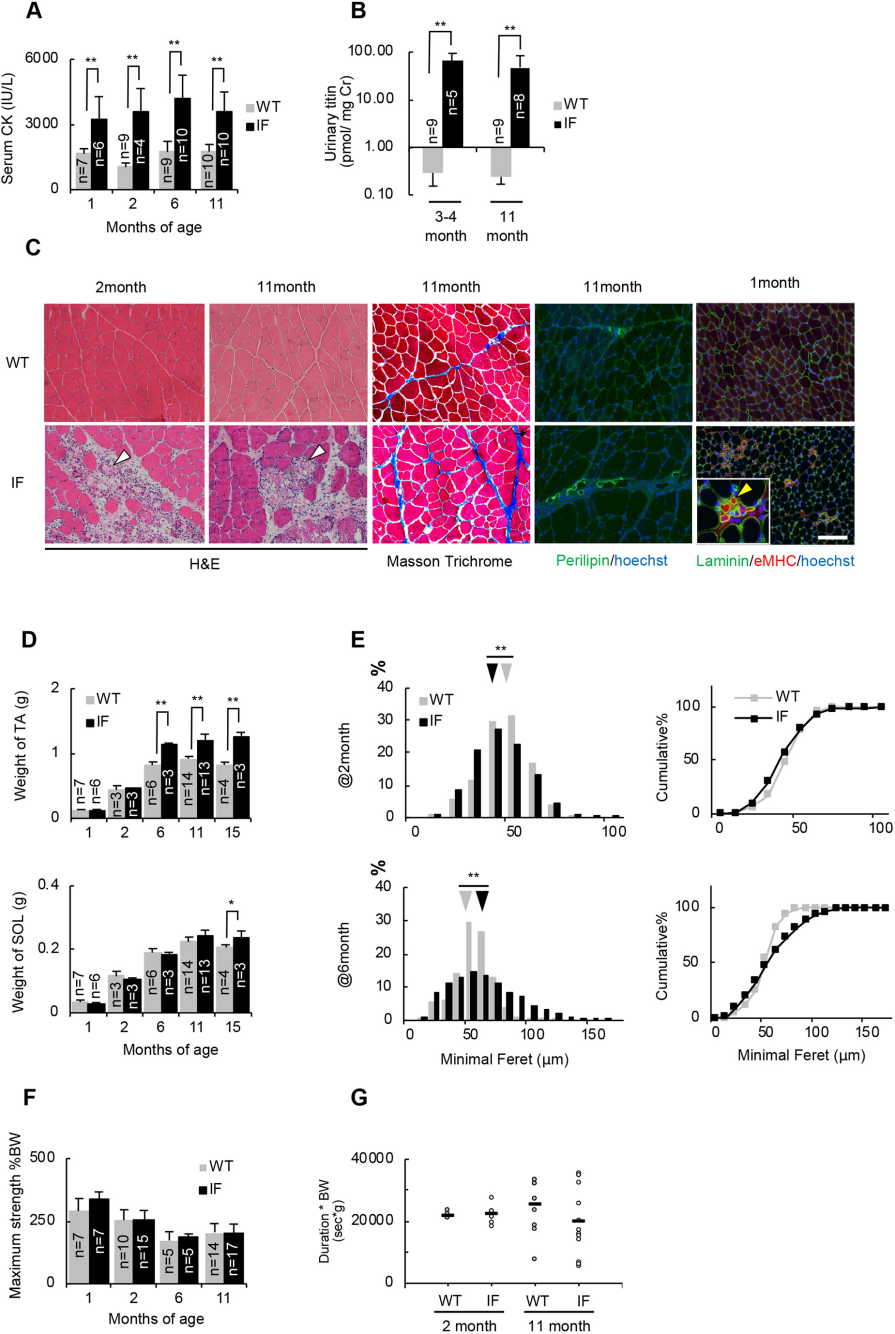
**Phenotypic analysis of** **the** **skeletal muscles of IF rats.** (A,B) Serum creatine kinase (A) and urinary titin (B) activity in wild-type (WT) and IF rats at different ages. Values of urine titin are standardized by the value of urinary creatinine. (C) Representative images of hematoxylin-eosin (H&E) staining, Masson’s trichrome staining, immunostaining of perilipin, and eMHC on TA muscle sections of wild-type and IF rats at different ages. White arrowheads indicate lesions with degenerated fibers and inflammatory cells. Yellow arrowhead indicates an eMHC^+^ regenerated fiber. Scale bar: 50 µm. (D) Weight of the TA and SOL of 1-, 2-, 6-, 11- and 15-month-old wild-type and IF rats. (E) Relative distributions and cumulative plots of myofiber size in the TA in 2- and 6-month-old wild-type and IF rats. Each arrowhead indicates the median value of each group. The data in each group contain the total number of detected myofibers from three subjects per group. (F) Maximum strength in ten trials of the grip test, standardized by body weight (%) at various ages. (G) Results of the four-limb hanging test in 2- and 11-month-old wild-type and IF rats. *n*=5-13, in each group. Bars represent the mean value of each group. Data are mean+s.d. (A,B,D,F). Statistical significance was determined using an unpaired two-tailed Student's *t*-test (A,B,D) or an unpaired two-tailed Wilcoxon rank sum test (E). *P<0.05, **P<0.01.

First, we compared two muscle injury markers of IF rats to age-matched wild-type controls. Serum creatine kinase (CK) is the most frequently used muscle injury marker. Additionally, titin fragment in urine has been described as a new non-invasive biomarker reflecting muscle tissue injury (Matsuo et al., 2019). In our study, both serum and urinary titin levels were significantly elevated in IF rats of all ages (Fig. 2A,B), indicating that skeletal and/or cardiac muscle were continuously affected.

Next, we analyzed the tissue pathology of skeletal muscles in IF rats. As fast-twitch myofibers are more affected in dystrophic muscles (Selsby et al., 2012; Webster et al., 1988), we focused on the tibialis anterior (TA) muscle, which is primarily composed of fast-twitch myofibers. Hematoxylin-eosin staining on TA muscle showed the appearance of lesions containing degenerated fibers and an invasion of inflammatory cells irrespective of animal age (Fig. 2C, Fig. S1A). In addition, the accumulation of intramuscular fibrous tissue was observed in all ages investigated (Fig. 2C, Fig. S2A,B) and intramuscular adipose tissue was observed at the later stage of the disease (11 months) (Fig. 2C, Fig. S2C). In addition, embryonic myosin heavy chain^+^ (eMHC^+^) regenerating fibers were present only in IF tissues (Fig. 2C, Fig. S1B) and the number of eMHC^+^ fibers per section decreased at 11 months of age (Fig. S1C), indicating that the regenerative capacity is kept until 6 months of age and decreases at 11 months of age. Next, to compare the number of myogenic cells contributing to muscle regeneration at 6 months of age to those at 11 months of age, we analyzed the number of Pax7(^+^) myogenic cells in primary culture derived from extensor digitorum longus (EDL) muscles of 6- and 11-month-old wild-type and IF rats (Fig. S1D). There was no significant difference in the number of Pax7(^+^) cells per well between the genotypes at 6 months, whereas the number was significantly lower in IF than wild type at 11 months, implying that the potential number of myogenic cells decreases in 11-month-old IF skeletal muscles. These results indicate that IF rats displayed typical dystrophic histopathological characteristics represented by muscle degeneration, followed by muscle regeneration, and fibrosis and adipose tissue infiltration. Moreover, the regenerative capacity decreases at 11 months, which might be caused by the decrease of myogenic cells.

Presumably, owing to continuous pathological changes, TA muscles of IF rats were significantly heavier than age-matched wild type at the late stage of disease (6, 11 and 15 months) (Fig. 2D), despite comparable body weights between genotypes (Fig. S3). To analyze whether muscle fiber hypertrophy occurred in TA muscle, we analyzed the distribution of minimal Feret diameter, which reflects the diameter of each muscle fiber (Briguet et al., 2004). At 2 months of age, the mean minimal Feret diameter of IF rats was significantly lower than in wild-type rats (Fig. 2E), indicating the appearance of small regenerating fibers. In contrast, at 6 months, the mean diameter was higher in IF rats (Fig. 2E). This might reflect the appearance of large regenerated myofibers with age, as observed in skeletal muscles of mdx mice (Sacco et al., 1992). To examine whether the dystrophic effect was also seen in slow-twitch fiber-rich muscle, the weight and distribution of the minimal Feret diameter of the soleus (SOL) muscle was analyzed. Our results showed that unlike TA muscle, the increase in weight was not observed in SOL muscle until 15 months of age (Fig. 2D). Moreover, the mean minimal Feret diameter of IF rats was significantly lower than in wild-type rats at 2, 6 and 11 months of age (Fig. S4), reflecting the appearance of small regenerating fibers. These results indicate that fast-twitch fibers were readily affected.

Next, to evaluate muscle strength *in vivo*, we conducted the grip test to evaluate forelimb strength, and the four-limb hanging test to evaluate whole-body motor skills, at different ages. We did not detect any significant difference in maximum strength in ten trials of the grip test at all ages (Fig. 2F). Furthermore, no significant difference was detected in the results from four-limb hanging tests between genotypes (Fig. 2G). Collectively, skeletal muscle of IF rats showed the dystrophic phenotype by way of muscle degeneration, increased interstitial fibrous/adipose tissue and muscle hypertrophy, but did not exhibit altered functional deficiency.

### IF rats display histopathological changes in cardiac muscles but not functional deficiency

Patients with BMD frequently display cardiac involvement, and cardiomyopathy is the number one cause of death for patients with BMD who live until the fifth or sixth decade of life (Finsterer and Stöllberger, 2003).

**Fig. 3.**
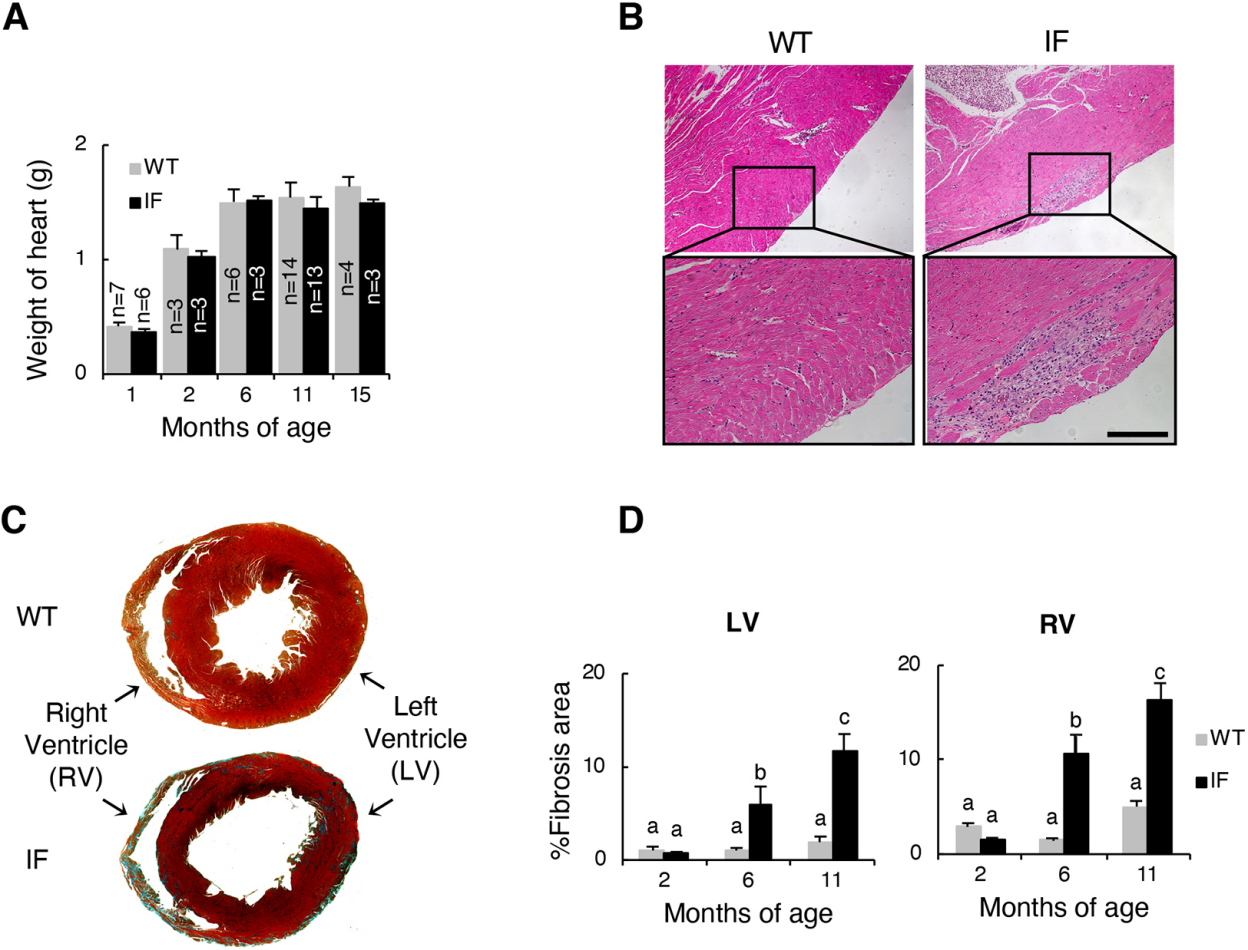
**Histopathology of cardiac** **muscles of IF rats.** (A) Weight of hearts of 1-, 2-, 6-, 11- and 15-month-old wild-type (WT) and IF rats. Data are means+s.d. (B) Representative images of hematoxylin-eosin staining of heart sections of 6-month-old wild-type and IF rats. Scale bar: 50 µm. (C) Representative images of Masson’s trichrome staining of hearts of 11-month-old wild-type and IF rats. (D) Quantitative analysis of fibrosis area in left and right ventricle wall of 2-, 6- and 11-month-old wild-type and IF rats (*n*=3 in each group). Different letters indicate significant differences between groups. Data are mean±s.d (A,D). Statistical significance was determined using an unpaired two-tailed Tukey's test (*P*<0.05).

To explore whether IF rats displayed cardiac failure, we conducted histopathological analyses of cardiac muscles and functional evaluation using echocardiography. The heart weight did not differ between genotypes at all ages investigated (Fig. 3A). However, in the cardiac muscle tissue of IF rats, inflammatory lesions were observed at all ages, indicating continuous cardiac muscle tissue degeneration (Fig. 3B, Fig. S4A). Furthermore, increased myocardial fibrosis was observed in cardiac muscles of IF animals (Fig. 3C). Also, the fibrotic area percentage increased with age, both in right and left ventricle (RV and LV, respectively) walls in IF animals, whereas there was not a significant change with age in wild type (Fig. 3D, Fig. S4B).

Subsequently, we evaluated cardiac function using echocardiography at 7 and 16 months (Table S1). Our results showed that the heart rates did not differ between genotypes. Functionality of the right and left sides of the heart of IF rats was evaluated by comparing echocardiographic variables with age-matched wild-type animals. Almost all left-heart variables investigated, such as wall thickness, LV diameters, LV fractional shortening, LV transmitral E and tissue Doppler imaging (TDI) peak systolic mitral annulus velocity (Sm) in septum, did not differ significantly between the genotypes. These results suggest that the tissue pathological changes do not result in obvious cardiac dysfunction in IF rats. In contrast, we observed significant differences in TDI Sm lateral wall at 16 months. This might reflect the promoted fibrotic lesion observed in the outer area of the left ventricular free wall of the IF rats. The LV diastolic index, TDI peak early diastolic mitral annulus velocity (Ea) and transmittal E/Ea were also evaluated but no significant difference was observed. Furthermore, there was no significant difference in the LV myocardial performance index that reflects both systolic and diastolic capacity, suggesting that the IF LV systolic capacity and diastolic capacity are relatively maintained in IF and wild-type animals.

No significant differences were observed between genotypes in all right heart variables analyzed. However, RV fractional area change, tricuspid annular plane systolic excursion (TAPSE) and Sm RV free wall, which are the indexes for RV systolic function, uniformly tended to decrease in 17-month-old IF rats. These results raise the possibility that, in particular, right heart dysfunction occurs in IF rats after 17 months of age, reflecting promoted fibrosis in the RV wall (Fig. S5A,B).

Collectively, the cardiac muscles of IF rats exhibited progressive histopathological changes represented by fibrosis. However, no obvious dysfunction was detected in most variables even at the later stage of the disease, although several variables showed a significant difference or declining trend.

### Expression of truncated dystrophin is significantly reduced in skeletal muscles of IF rats

**Fig. 4.**
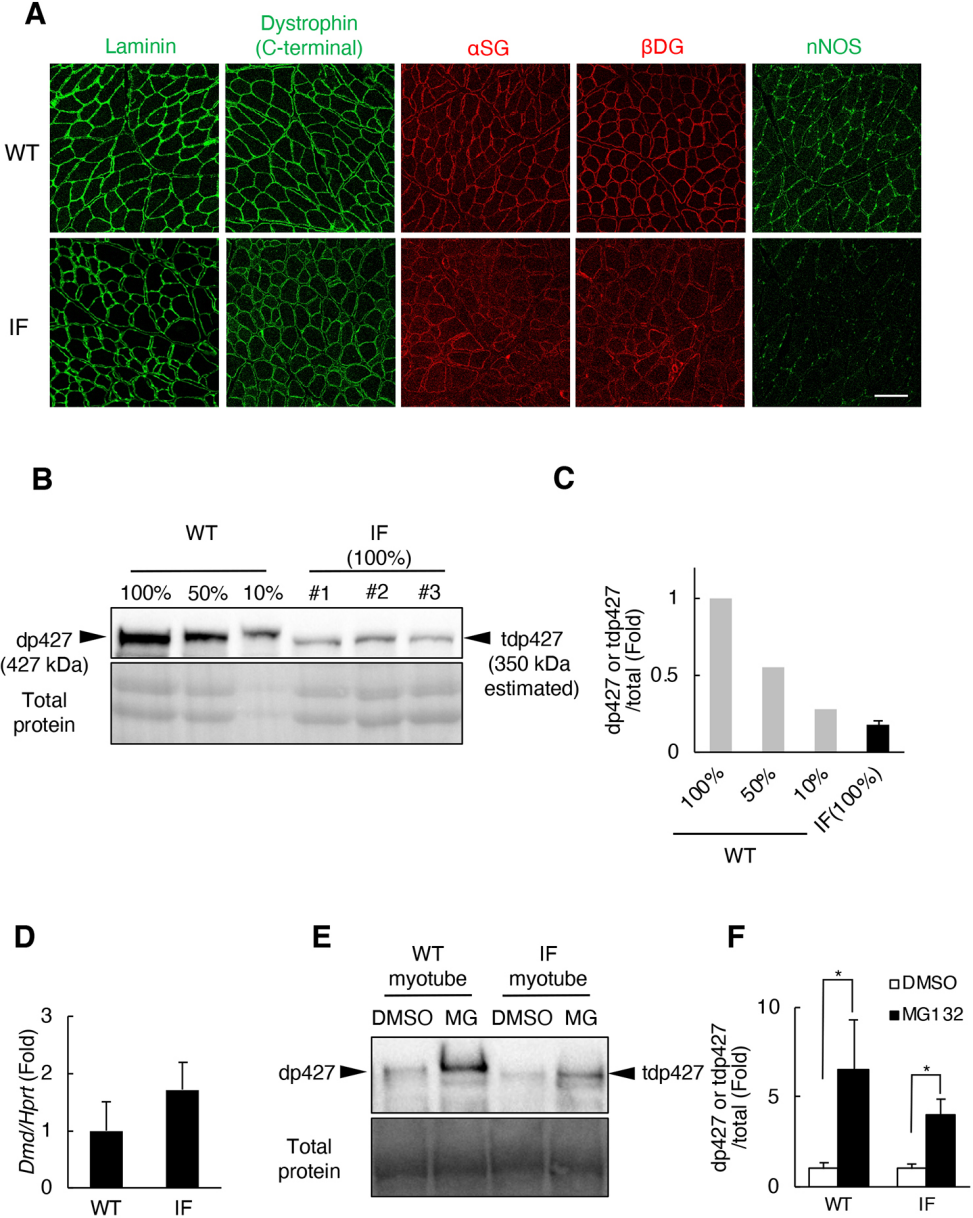
**Expression of truncated dystrophin and DGC components in the skeletal muscle of IF rats.** Immunohistochemical staining of laminin, dystrophin and other DGC components in TA sections of 11-month-old wild-type (WT) and IF rats. Dystrophin was detected with anti-dystrophin (rabbit monoclonal) antibody. Scale bar: 50 µm. (A) Immunoblot analysis of dp427/tdp427 protein expression in the TA of 1-month-old wild-type rats at three dilutions, and of age-matched IF rats. Ponceau S staining was used as a loading control. (B,C) Immunoblot analysis (B) of dp427/tdp427 expression levels (quantified in C). IF, *n*=3. (D) *Dmd* mRNA levels standardized by *Hprt* in the TA of 1-month-old wild-type and IF rats. (wild type, *n*=3; IF, *n*=6). (E) The effect of proteasome inhibitor (MG132) treatment on the expression of dp427/tdp427 protein in myotubes derived from wild-type/IF skeletal muscles. Dystrophin was detected with anti-dystrophin (rabbit monoclonal) antibody. Ponceau S staining was used as a loading control. (F) dp427/tdp427 expression levels quantified by immunoblot analysis shown in E. Data are mean+s.d. (D,F). IF: *n*=3. Statistical significance was determined using an unpaired two-tailed Student's *t*-test. *P<0.05.

In-frame mutations in *DMD* generally result in decreased expression of tdp427 and DGC components compared with healthy controls (Beggs et al., 1991). We determined the expression of dystrophin and components of the DGC (αSG, βDG and nNOS) in skeletal muscles of IF rats using immunohistochemistry and immunoblot analyses. Immunostaining on TA muscles showed that in both wild-type and IF rats, the dystrophin and DGC^+^ regions were localized beneath the sarcolemma (Fig. 4A). Immunoblot analysis showed that in IF skeletal muscles full-length dystrophin was truncated to a lower molecular weight (∼350 kDa) based on genetic mutations (Fig. 4B). In addition, expression of truncated dystrophin was reduced to around 10% of wild type despite adequate gene expression (Fig. 4C,D). Immunoblot analysis showed that expression levels of βDG were significantly reduced and those of αSG were slightly reduced in IF skeletal muscles (Fig. S6).

Next, to examine whether the decrease of tdp427 is mediated by enhanced proteasomal degradation, myotubes derived from wild-type and IF rats were treated with the proteasome inhibitor MG-132 *in vitro*, and the protein levels of dp427/tdp427 were measured using immunoblot analysis. As proteasome inhibition completed, dp427/tdp427 levels were recovered (Fig. 4E); moreover, no enhanced protein recovery was observed in IF myotubes compared with wild-type myotubes (Fig. 4F), suggesting that the expression level of both dp427 and tdp427 was controlled by proteasomal degradation, but a different mechanism was responsible for the reduction of tdp427 in IF skeletal muscles.

These results indicate that in-frame deletion in *Dmd* results in the expression of truncated dystrophin and the reduction of truncated dystrophin and DGC components in skeletal muscle tissues. Moreover, *in vitro*, the reduction of tdp427 is not caused by enhanced proteasomal degradation.

### Additional dystrophin is specifically expressed in skeletal/cardiac muscle tissues of IF rats

**Fig. 5.**
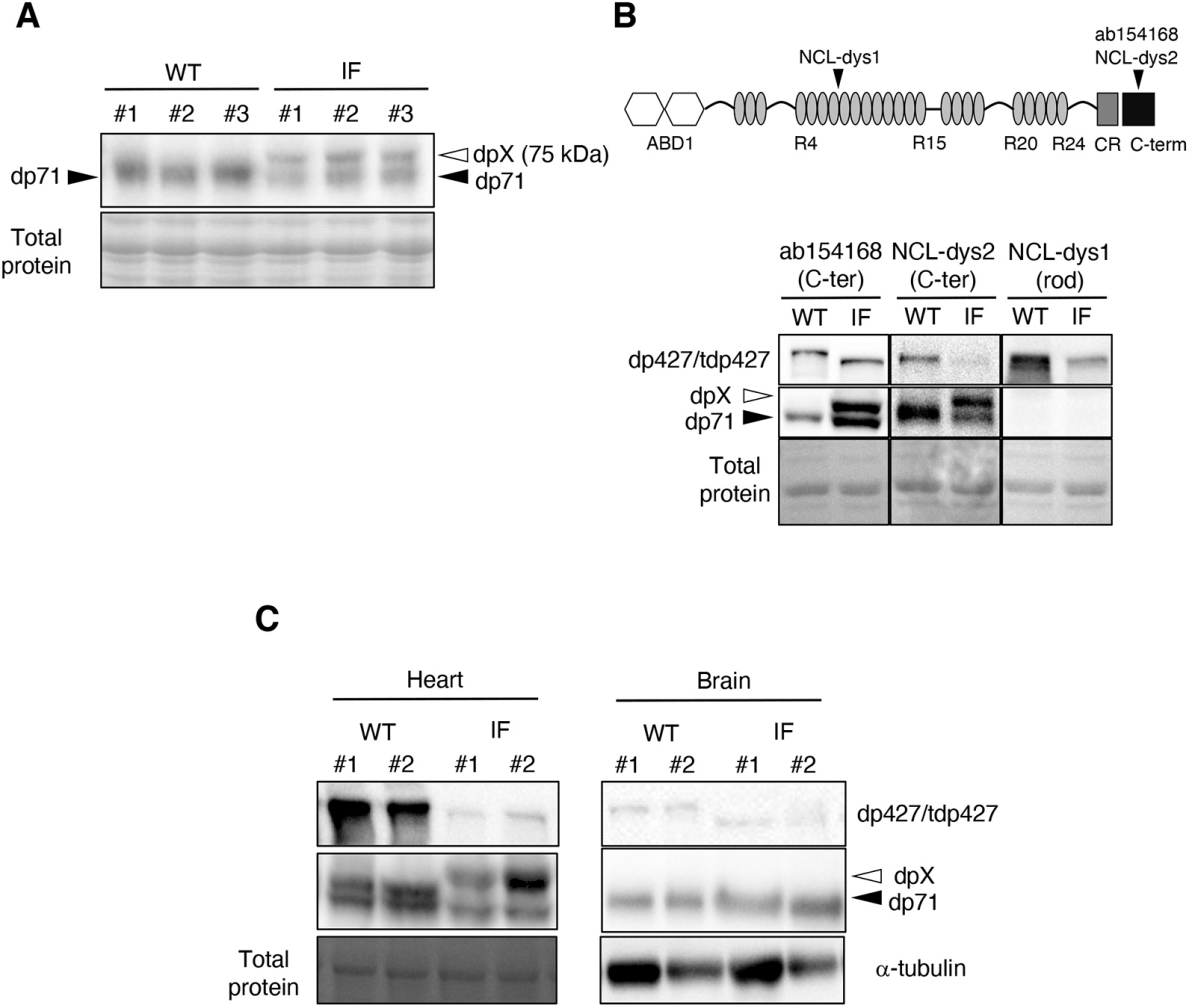
**Expression of additional dystrophin (dpX) in IF rats.** (A) Immunoblot analysis on the lysates from the TA of 1-month-old wild-type (WT) and IF rats. Ponceau S staining was used as a loading control. (B) Comparative immunoblot analysis with different anti-dystrophin antibodies on the lysates from TA of 1-month-old wild-type and IF rats. The blots were analyzed with antibodies against the C-terminus (ab154168, NCL-dys2) and rod domain (NCL-dys1) of full-length-dystrophin. Ponceau S staining was used as a loading control. The schematic represents the composition of full-length dystrophin and the sites of the immunogen of three antibodies on it. (C) Immunoblot analysis on the heart and brain lysates of 1-month-old wild-type and IF rats. Dystrophin detection was performed with anti-dystrophin antibody (ab154168). Ponceau S staining (heart) and α-tubulin (brain) were used as a loading control, respectively.

Mutations in *DMD* not only trigger abnormal expression of dp427, but on occasion result in the appearance of unidentified proteins, such as novel dystrophin isoforms (Wein et al., 2014) or additional dystrophin, which is possibly a degradation product of dp427 (Beggs et al., 1992; Hoffman et al., 1988). To explore whether such unidentified proteins appear in IF skeletal muscles, we performed immunoblotting experiments with anti-dystrophin antibody and focused on the detected bands with a molecular weight lower than 427 kDa. As a result of immunoblot analysis with anti-C-terminal domain of dystrophin antibody, 71 kDa dystrophin isoform (dp71), which is a ubiquitously expressed isoform (Kawaguchi et al., 2018; Tadayoni et al., 2012), was detected in skeletal muscle lysate of both genotypes (Fig. 5A). Additional 75 kDa signals (dpX) were detected in IF rats, but not in wild-type rats (Fig. 5A). To predict the molecular structure of dpX, comparative immunoblot experiments were performed using different anti-dystrophin antibodies. dpX was detected with another anti-C-terminal domain antibody (NCL-dys2) but not with anti-rod domain antibody (NCL-dys1) (Fig. 5B), indicating that dpX possesses the C-terminal structure of dp427 but lacks the proximal rod domain. These results suggest that dpX possesses a partial structure of dp427 and is possibly an unidentified novel dystrophin isoform or a degradation product of tdp427. dp427 is tissue-specifically expressed in skeletal/cardiac muscle and brain (Blake and Kro˚ger, 2000; Nudel et al., 1989). Thus, we next explored whether dpX is present or not in cardiac muscle and brain, namely, tissues expressing dp427 other than skeletal muscle. We detected dp427/tdp427 expression both in cardiac muscle and brain, whereas dpX was only detected in cardiac muscles of IF rats (Fig. 5C). These results indicate that dpX is expressed only in tdp427-expressing muscle tissues.

### dpX connects to βDG on the sarcolemma

**Fig. 6.**
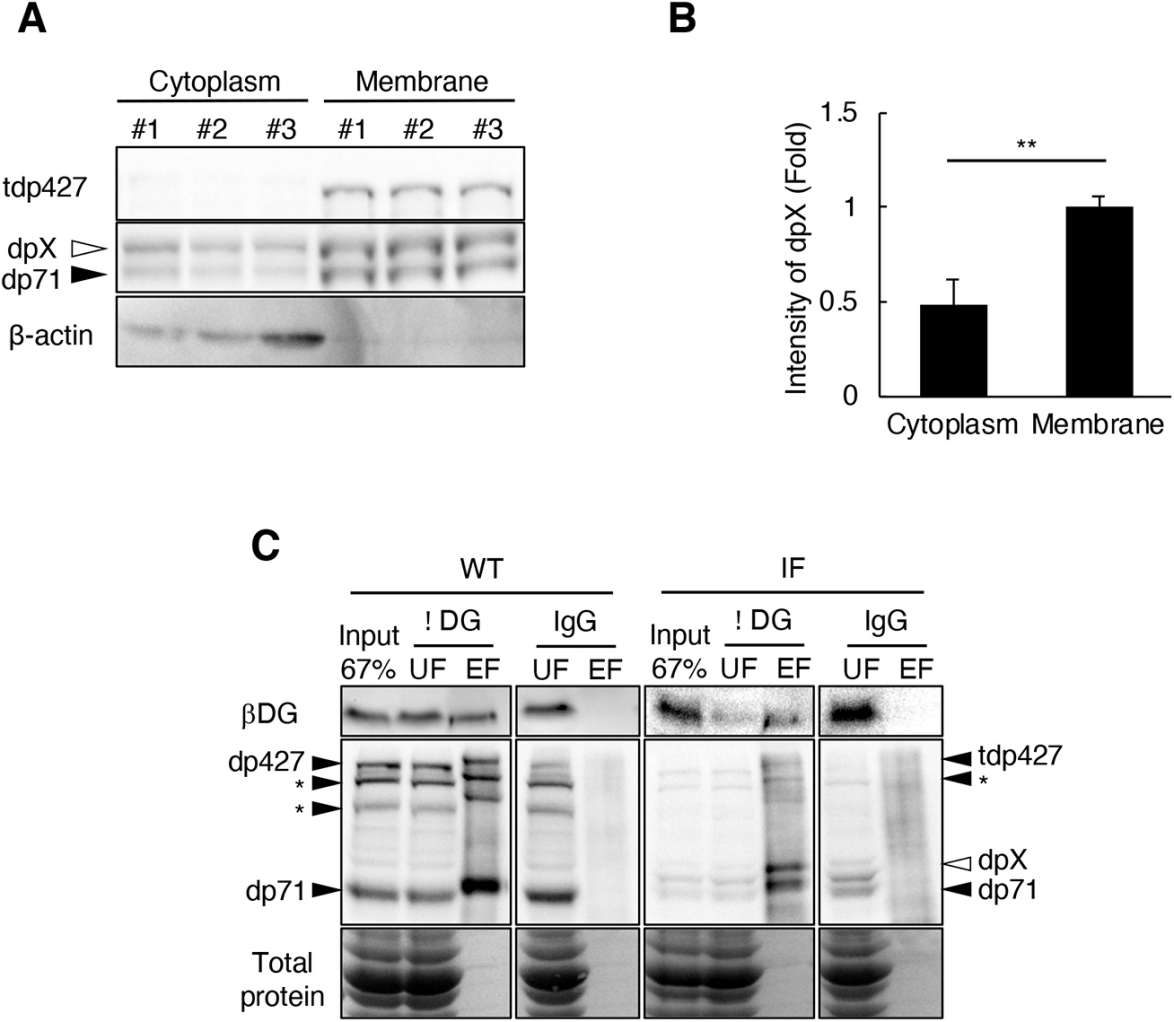
**Membrane-localization of dpX.** (A) Immunoblot analysis with anti-dystrophin antibody on the cytoplasm and membrane fraction of skeletal muscles of IF rats. Equal amounts of protein were applied to each well. β-actin was used as a cytoplasm marker. (B) Relative intensity of dpX in the cytoplasm and membrane fraction shown in A. Data are mean+s.d. (*n*=3, in each group). Statistical significance was determined using an unpaired two-tailed Student's *t*-test. **P<0.01. (C) Immunoblot analyses on immunoprecipitated fractions with anti-βDG antibody (7D11) and with an isotype matched control antibody (IgG) as a control. The immunoprecipitation was performed on the membrane preparation from TA of 1-month-old wild-type (WT) and IF rats. The blots were analyzed with 7D11 and anti-dystrophin antibody. EF, elution fraction; UF, Unbound fraction. Asterisks indicate unknown additional bands. Ponceau S staining was used as a loading control.

The C-terminal structure of dp427 is essential for DGC formation mediated by binding with a transmembrane DGC component, βDG (Rentschler et al., 1999). Thus, we performed DGC pulldown using immunoprecipitation with anti-βDG antibody to examine whether dpX contributes to DGC formation. Before this experiment, we confirmed that most dpX was included in the membrane fraction (Fig. 6A,B) using an immunoblot experiment. The pulldown analysis revealed that dp427 in wild type and tdp427 in IF co-immunoprecipitated with βDG using the anti-βDG antibody (Fig. 6C), indicating that in-frame mutations affecting N-terminal regions do not necessarily affect dp427-βDG binding. In addition, dpX co-immunoprecipitated with βDG, indicating that dpX binds to βDG directly and is a component of the DGC (Fig. 6C). Collectively, these results imply that dpX is a newly discovered DGC component in the skeletal muscles of IF rats.

## DISCUSSION

In this study, we established a novel rat model that carries in-frame mutations in *Dmd* (IF rats) and exhibits reduced expression of truncated dystrophin. Our results showed that the abnormal dystrophin expression resulted in the dystrophic changes in muscle tissues but did not lead to functional deficiency. We also found that the expression of dpX, which forms the DGC in the sarcolemma, was associated with the appearance of tdp427.

Expression of tdp427 in muscle tissues is one of the factors that determine severity of BMD (Anthony et al., 2011; Van Den Bergen et al., 2014). Reports using *in vitro* or transgenic mice models have shown that the point mutations in the 5′ region of *DMD* result in the ubiquitin-proteasome system-mediated degradation of mutated dp427 (Singh et al., 2010; Talsness et al., 2015). However, our study demonstrated that although the expression level of tdp427 was significantly reduced in skeletal muscles, the proteasomal degradation was not enhanced when compared to dp427 *in vitro*. These results indicate that the difference in the instability of tdp427 and dp427 is not due to the change in tertiary structure. Therefore, a different mechanism might underlie the decrease in the tdp427 levels. However, it should be noted that the proteasomal inhibition in this study occurred in the limited *in vitro* situation. Further research is needed to clarify the mechanism underlying the decrease of tdp427 in IF muscle tissues *in vivo*. A previous study has reported that TNFα-induced microRNAs control dystrophin expression in BMD (Fiorillo et al., 2015). As continuous inflammation was observed in IF skeletal muscles, it is possible that the inflammation-related microRNAs contribute to the decrease of tdp427. Furthermore, several reports have indicated that the forced expression of dystrophin isoforms (dps), which have the same C-terminal structure as dp427, can compete with dp427 for sarcolemma binding, thereby decreasing the levels of dp427 in wild-type skeletal muscles (Leibovitz et al., 2002; Warner, 2002). In this study, we demonstrated that dpX contained the C-terminal structure of dp427, and both tdp427 and dpX, localized in sarcolemma and formed DGC. Therefore, dpX, as well as microRNA, might contribute to the decrease of tdp427 in the skeletal muscles of IF rats by competing with tdp427 on the sarcolemma in the same manner as dps.

Our results did not elucidate the mechanism underlying the disease-specific expression of dpX in IF muscle tissues. However, according to the previous reports, the expression of dpX might be regulated at pre- and/or at post-transcriptional levels of *Dmd* mRNA. The retention of intronic sequence in mature *DMD* mRNA has been reported in patients with Duchenne muscular dystrophy (Nishida et al., 2015). In addition, mutations in *DMD* occasionally result in the appearance of novel dystrophin isoforms caused by the alternative translation initiation (Wein et al., 2014). Similar to these reports, *Dmd* mutation in IF rats possibly causes the intron retention in *Dmd* mRNA or the alternative translation initiation. Also, intron retention might trigger the unpredicted splicing of *Dmd* mRNA, which leads to the expression of dpX in the muscle tissues of IF rats. Furthermore, it has been reported that skeletal muscle biopsies from human patients with BMD, whose mutations are located in the distal rod domain (exons 45 to 53), show additional dystrophin expression (Beggs et al., 1992; Hoffman et al., 1988). The authors concluded that the additional dystrophin is a proteolytic product of tdp427. In this study, we demonstrated that dpX contained the C-terminal structure of dp427 and dpX expression was restricted to tdp427-expressing tissues, except for the brain. These results indicate that dpX could be a breakdown product of tdp427, similar to additional human dystrophin. A previous *in vitro* study demonstrated that calcium-dependent proteases, calpain I and II, cleave dp427 into products of different sizes (Cottin et al., 1992). It is possible that in the muscle tissues of IF rats such proteases are more activated than in that of healthy controls, as reported in the skeletal muscles of mdx mice (Gailly et al., 2007). Additionally, the function of dpX in muscle tissues also remains unknown. In this study, we demonstrated that both tdp427 and dpX localized in the sarcolemma and formed DGC. As dp71-mediated DGC restoration did not ameliorate the pathology of mdx mice (Cox et al., 1994), it is conceivable that dpX, which might have a similar molecular weight and structure as dp71, also does not play a big ameliorating role in IF muscle tissues. Collectively, our data imply that the endogenous additional dystrophin, such as dpX, the expression of which is triggered by in-frame mutations in *Dmd*, might restore DGC on the sarcolemma. Further research is required to reveal the mechanism underlying the appearance and function of dpX.

In our previous study, the dystrophin deficient (Duchenne muscular dystrophy) rats exhibited muscle tissue pathology similar to that of muscular dystrophy, exhibiting continuous muscle degeneration and deposition of intramuscular fibrous tissues and fat. Furthermore, the animals showed a noticeable decrease in muscle strength at 13 weeks of age (Nakamura et al., 2014). In contrast, the IF rats did not show any detectable impaired function in the skeletal and cardiac muscles, despite the presence of tissue pathological changes similar to Duchenne muscular dystrophy rats. Furthermore, in muscle tissues, we confirmed the expression of tdp427 that lacked the region corresponding to the mutated DMD protein. These findings imply that tdp427-lacking domains, encoded by exons 3 to 16, do not sufficiently function as sarcolemma-supporting proteins to prevent histological lesions, but are sufficiently functional to ensure normal force generation by muscle tissue. Recently, the role of dystrophin in neuromuscular junctions (NMJs) has become clearer. A NMJ is a specialized synapse formed between the terminal end of a motor neuron and a muscle fiber to transmit the nerve impulses to the muscle. The impaired function of muscle tissues, represented by the decrease in muscle strength and easy fatigability, is considered to be triggered by NMJ abnormalities. It is reported that the dystrophin is enriched post-synaptically at NMJs and aids neuromuscular transmission (Bewick et al., 1992). The absence of dystrophin at NMJs in mdx mice causes neuromuscular transmission defects that aggravate muscle weakness (van der Pijl et al., 2016). Although it remains unclear which dystrophin domain is important for NMJ functionality, the tdp427 in IF rats, even with a missing part of the protein, might be sufficient for normal NMJ function. This might explain the lack of functional deficiency in IF rats.

In conclusion, this study demonstrated the usefulness of the novel rat BMD model in reflecting the tissue pathology associated with human BMD. Furthermore, our results provide the expression profile of novel dystrophin-related product (dpX) in BMD with mutations within the 5′ region of the *DMD* gene. Although the IF rats described in this study do not entirely cover the overall pathology seen in human BMD, in which variable pathological states are observed among individuals, the outcome of the study furthers our understanding of BMD pathology, and indicates the possibility of dystrophin recovery treatments for patients with Duchenne muscular dystrophy.

## MATERIALS AND METHODS

### Animals

A rat strain carrying in-frame mutations in the *Dmd* gene (IF strain) was maintained in our laboratory under controlled environmental conditions: 23°C with a photoperiod of 12-h light and 12-h dark (lights on at 0800 h). This strain was derived from a female rat that was a littermate of *Dmd*-mutated F0 rats reported previously (Nakamura et al., 2014). The female IF rat was mated with the male Wistar–Imamichi strain, purchased from the Institute for Animal Production, to maintain the strain. IF and age-matched wild-type males were used in this study. Animals were fed commercial chow *ad libitum* (Lab MR-Breeder Standard, Nihon Nosan Kogyo). All animal experiments in this study were performed in accordance with the Guide for the Care and Use of Laboratory Animals of the University of Tokyo and were approved by the Institutional Animal Care and Use Committee of the University of Tokyo (certificate number P18-125).

### RT- PCR

After TA muscles were homogenized using a Shake Master (ver. 1.0, Bio Medical Science Inc), RNA was isolated using TRIzol (Invitrogen) and reverse-transcribed to cDNA using SuperScriptII reverse transcriptase (Invitrogen). qRT-PCR was performed using a Light Cycler 2.0 (Roche) with Thunderbird SYBR qPCR Mix (Toyobo). The sequences of the primer sets used in RT-PCR and qPCR are listed in Table S2.

### Sequencing analysis

Genomic DNA and cDNA were obtained from TA muscles. PCR was performed to confirm the deletion of the *Dmd* gene and *Dmd* mRNA. PCR products were purified by agarose gel electrophoresis and subsequently sequenced as reported previously (Fujii et al., 2013).

### Histological analyses

Histological analyses were performed using a method published previously (Nakamura et al., 2014). Briefly, frozen sections (7 μm) of TA muscles were prepared transversely, and paraffin-embedded sections of the heart were subjected to histological analyses. The sections were used for hematoxylin and eosin staining and Masson's trichrome staining, then were dehydrated and mounted.

For immunostaining, cryosections were fixed with 4% paraformaldehyde. After blocking with 5% normal donkey serum in PBS containing 0.1% Triton X-100 (Sigma-Aldrich), cryosections were incubated overnight with primary antibodies (described below) at 4°C, followed by washing and incubation with AlexaFluor-conjugated secondary antibodies (1:500, Jackson ImmunoResearch) for 1 h. Nuclei were counterstained with Hoechst 33258. For quantitative analyses of the fibrotic area, five to 20 fields were randomly selected in the sections with Masson’s trichrome staining using a 10× objective for skeletal muscles and a 4× objective for cardiac muscles. The area occupied by fibrotic tissues stained blue and the total area of sections were calculated using ImageJ software. For quantitative analyses of myofiber diameters, ten fields in TA and five fields in SOL were randomly selected in the sections stained with anti-laminin antibody using a 10× objective. The minimal Feret's diameter was calculated using ImageJ software. For quantitative analyses of eMHC^+^ fibers, the number of positive fibers per section was counted using a microscope. Photos were taken using a BX51 fluorescence microscope (Olympus) equipped with a DP73 digital camera (Olympus).

Primary antibodies and their species of origin were as follows: anti-dystrophin (rabbit monoclonal, ab154168, 1:400); anti-laminin (rabbit polyclonal, L9393, 1:100, Sigma-Aldrich); anti-eMHC (mouse monoclonal, F1.652, 1:100, Developmental Studies Hybridoma Bank); anti-α-sarcoglycan (mouse monoclonal, AD1/20A6, 1:100, Novocastra); anti-β-dystroglycan (mouse monoclonal, 43DAG1/8D5, 1:100, Novocastra); and anti-nNOS (rabbit polyclonal, 1:100, Invitrogen).

### Immunoblotting

TA muscles were lysed in radioimmunoprecipitation assay buffer [50 mM Tris-HCl (pH 7.4), 1% NP-40, 0.5% Na-deoxycholate, 0.1% SDS, 150 mM NaCl, 2 mM EDTA and 50 mM NaF]. For perilipin detection, sample buffer (0.5 M Tris-HCl, 10% glycerol, 1% SDS and 10% β-mercaptoethanol) was used during homogenization. Protein concentration was determined using the bicinchoninic acid assay reagent (Fujifilm-WAKO). Equal amounts of protein were resolved on a 10% polyacrylamide gel and subsequently transferred onto a polyvinylidene difluoride membrane (Millipore). The membranes were blocked with 5% skimmed milk in Tris-buffered saline containing 0.1% Tween 20 and incubated with primary antibodies (described below) overnight at 4˚C, followed by incubation with horseradish peroxidase-conjugated secondary antibodies (1:5000) for 1 h at room temperature. Protein bands were detected using enhanced ECL prime (GE Healthcare) and a ChemiDoc (Bio-Rad). The band intensity was determined using Image Lab software (Bio-Rad).

Primary antibodies used in the experiments were as follows: anti-dystrophin (rabbit monoclonal, ab154168, 1:400, Abcam); NCL-dys1 (mouse monoclonal, 1:100, Novocastra); NCL-dys2 (mouse monoclonal, 1:100, Novocastra); anti-α-sarcoglycan (mouse monoclonal, AD1/20A6, 1:100, Novocastra); anti-β-dystroglycan (mouse monoclonal, 7D11, 1:400, Santa Cruz Biotechnology), anti-perilipin (rabbit monoclonal, D1D8, 1:1000, Cell Signaling Technology); anti-α-tubulin (rabbit polyclonal, ab4074, 1:660, Abcam); and anti-β-actin (rabbit monoclonal, 13E5, 1:500, Cell Signaling).

### Creatine kinase activity

Whole blood was collected from abdominal aorta under anesthesia with isoflurane and spun at 1000 ***g*** for 30 min at 4°C. Creatine kinase activity in separated sera was assayed using the Fuji Dri-Chem system (Fujifilm).

### Urinary titin

Urine samples were obtained from 3-, 4- and 11-month-old IF rats and age-matched wild-type rats. Urinary titin was measured using an ELISA system using the Titin-N Fragment Assay Kit-IBL (Immuno-Biological Laboratories) (Matsuo et al., 2019), as described previously (Awano et al., 2018). Urinary creatinine concentrations were measured using an assay kit (LabAssay Creatinine). This kit detects titin-N fragments of skeletal and cardiac muscles (Yoshihisa et al., 2018).

### Skeletal muscle function

#### Grip test

The grip test was performed to evaluate the strength of forelimb muscles. The method complies with the modified version of ‘Use of grip strength meter to assess the limb strength of mdx mice’ (TREAT-NMD SOP ID: DMD_M.2.2.001) (Bladen et al., 2015). Briefly, rats were placed with their forelimbs on a T-shaped bar and were gently pulled backward until they released their grip. A grip meter (GPM-101B, Melquest) measured the peak force generated. Ten tests were performed in sequence with a short latency between each test, and the maximum strength in ten trials was taken as an index of grip strength. Results are expressed as a percentage of the body weight (g/g).

#### Four limb hanging test

The four limb hanging test was performed to evaluate the strength of four limb muscles. The method complies with the modified version of ‘The use of four limb hanging tests to monitor muscle strength and condition over time’ (TREAT-NMD SOP ID: DMD_M.2.1.005; www.treat-nmd.org/wp-content/uploads/2016/08/MDX-DMD_M.2.1.005.pdf). Briefly, each rat was allowed to use its four limbs to grasp an aluminum mesh (35 cm×45 cm, 1.5 cm×1.5 cm square grid), which was placed vertically 1 m above a cushion. The time to fall was recorded and the value was multiplied by the body weight as an indication of muscle strength. A fixed limit was applied in hanging time (a maximum of 60 s). The records of rats who climbed over the top of the aluminum mesh and stayed still there were omitted from the data.

### Echocardiography

Echocardiographic images were acquired using a Vivid E95 digital ultrasound system (GE Vingmed Ultrasound) with a 12 MHz 12S-D probe (GE Vingmed Ultrasound) on 7- and 16-month-old wild-type and IF rats. Echocardiographic images were analyzed with EchoPAC (GE Healthcare). Rats were anaesthetized with isoflurane at a concentration of 5%. Rats were then placed on a heated platform, and anesthesia was maintained by 3-4% isoflurane. Under anesthesia, echocardiographic images were acquired. Images of the parasternal short-axis view at the papillary muscle level and the apical four-chamber view were recorded to obtain a higher frame rate (∼200 frames per second) with higher imaging qualities. For assessing LV diastolic function, early diastolic velocity peak (E) and late diastolic peak (A) were measured along the short-axis view using tissue Doppler mode. A wave was not detectable in this study. For assessing LV systolic function, fractional shortening and ejection fractions were calculated by measuring intraventricular septum thickness in diastole, LV posterior wall thickness, LV end-diastolic and end-systolic diameters. These values were measured along the parasternal short-axis view in motion mode (Mmode). LV myocardial performance index (MPI), a universal value of ventricular systolic and diastolic function, was determined using tissue Doppler mode. For calculating MPI, aortic ejection time and the non-flow time of the LV were measured. Fractional area change and TAPSE were evaluated as variables for RV function.

### *In vitro* myogenic cell isolation: analysis of myogenic cell numbers or proteasome inhibition

Skeletal muscle myogenic progenitor cells were obtained from the muscles of wild-type and IF rats as described previously (Yamanouchi et al., 2007). Briefly, muscles were hand minced using scissors, and digested for 1 h at 37°C with 1.25 mg/ml protease (from *Streptomyces griseus*, type XIV; Sigma-Aldrich). Cells were separated from muscle-fiber fragments and tissue debris through differential centrifugation, and plated on poly-L-lysine- and fibronectin-coated plates in 10% fetal bovine serum (FBS)/Dulbecco's modified eagle medium (DMEM). As for the analysis of myogenic cell numbers, EDL muscles of 6- and 11-month-old wild-type and IF rats were used. Cells separated from one side of EDL muscles were dispensed into two wells of a 48-well-plate. After a 2-day culture in 10% FBS/DMEM, cells were fixed with 4% paraformaldehyde, blocked with 5% normal goat serum in PBS containing 0.1% Triton X-100, and incubated overnight with anti-Pax7 antibodies (mouse monoclonal, P3U1, 1:100, Developmental Studies Hybridoma Bank) at 4°C and then for 1 h with AlexaFluor-conjugated secondary antibodies (1:500, Invitrogen). As for the proteasome inhibition, back and hindlimb muscles of neonatal rats (day 0-1) were used. After a 7-day culture in 10% FBS/DMEM, the proteasome inhibitor MG-132 (Enzo Life Sciences) or DMSO as control were added at a concentration of 5 μg/ml to the media for wild-type and IF myotubes differentiated from obtained myogenic progenitor cells. Total protein lysate was extracted with sample buffer after 24 h culture with MG-132/DMSO.

### Separation of membrane fraction

One half of frozen TA muscle was homogenized in ice-cold homogenization buffer [10% Sucrose, 0.5 mM EDTA, 1 mM phenylmethylsulfonyl fluoride (pH 7.2) and 0.2% protein inhibitor cocktail (Promega)] using a tissue homogenizer (Kinematica Polytron). Samples were then centrifuged at 12,000 ***g*** for 10 min at 4°C and the supernatant was discarded. To extract the membrane fraction, pellets were resuspended in extraction buffer [50 mM Tris-HCl (pH 7.4), 150 mM NaCl, 0.05% NP-40, 1% digitonin and 0.2% protein inhibitor cocktail] for 1 h on ice and then centrifuged at 20,000 ***g*** for 30 min at 4°C to remove the insoluble residue.

### Co-immunoprecipitation

The separated membrane fraction was pre-cleared of mouse antibody by incubation with protein G agarose beads (sc-2002, Santa Cruz Biotechnology) for 30 min at 4°C, followed by centrifugation at 5000 ***g*** to remove resin. To conjugate protein G with antibodies, protein G agarose beads (50 μl, sc-2002, Santa Cruz Biotechnology) were incubated with anti-βDG antibody (5 μg for βDG immunoprecipitation, 7D11, Santa Cruz Biotechnology) and isotype matched mouse IgG (5 μg for control immunoprecipitation, G3A1, Cell Signaling Technology) in PBS overnight at 4°C. Thereafter, the beads were washed three times with PBS. Next, 200 μg of pre-cleared protein lysate was added to 7D11- or G3A1-conjugated protein G agarose beads and the mixture was incubated overnight at 4°C in end-over-end mixing. After the incubation, supernatant was collected as unbound fraction. Beads were washed three times with PBS. Sample buffer was used for elution.

### Statistical analyses

A Student's *t*-test and one-way ANOVA followed by Tukey–Kramer's test were used to examine statistical differences between two groups and more than two groups, respectively. For the distribution of myofibers, median values were compared using the Wilcoxon rank sum test. *P*<0.05 was considered statistically significant. Graphed data represent mean+s.d.

## Supplementary Material

Supplementary information
