# Supplementary material for: Pathological evaluation of rats carrying in-frame mutations in the dystrophin gene: a new model of Becker muscular dystrophy
Source: Dis Model Mech. 2020 Sep 28;13(9):dmm044701. doi: 10.1242/dmm.044701 (PMC7541341; doi:10.1242/dmm.044701)
Supplement: Supplementary information [file dmm-13-044701-s1.pdf]

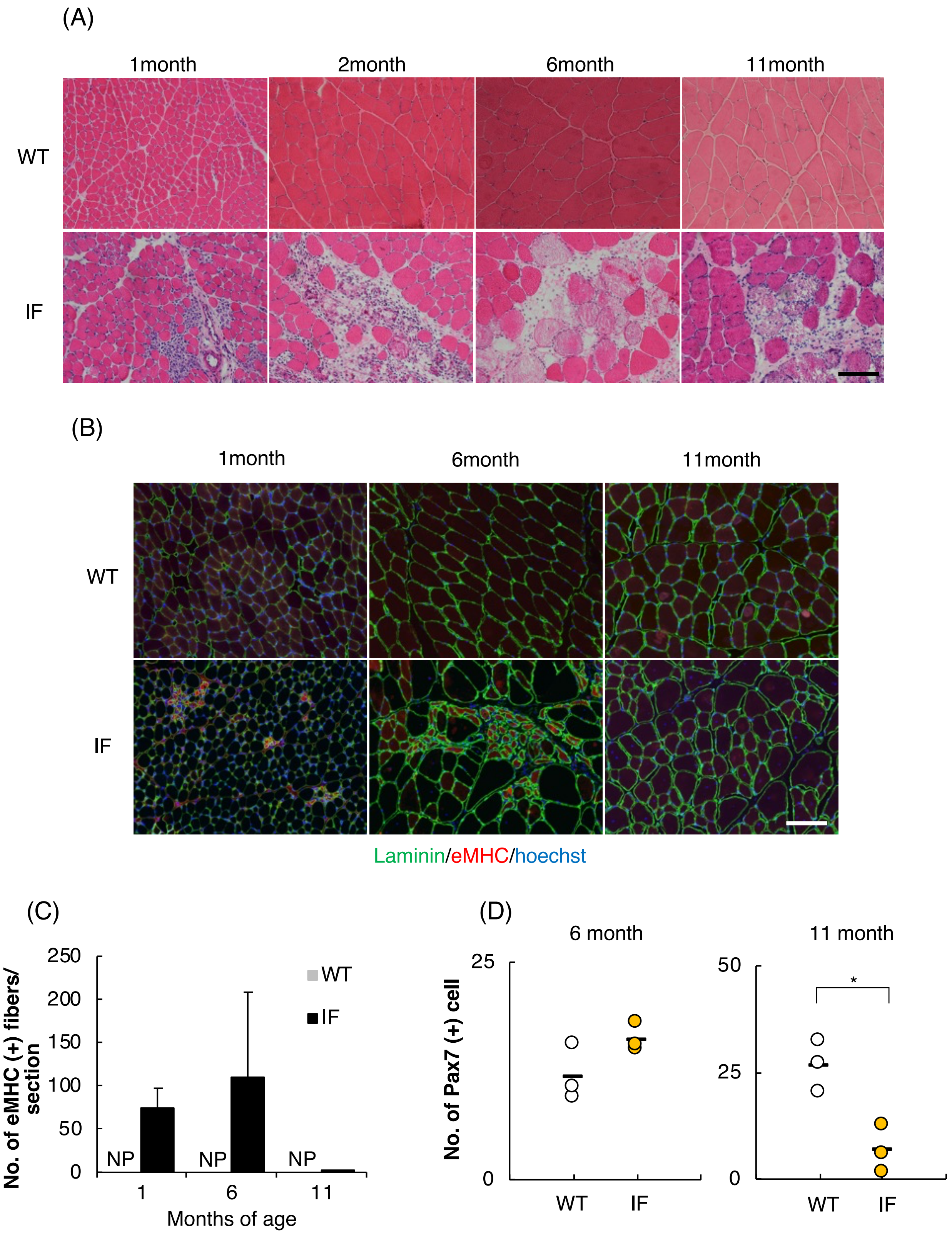

Figure. S1 Continuous degeneration and age-related change of regeneration capacity in skeletal muscles of IF rats.

(A) Representative images of H&E staining on TA muscle sections of 1, 2, 6, 11-month-old WT and IF rats. Scale bar = 50 μm.

(B) Representative images of immuno-staining for eMHC on TA muscle sections of 1, 6, 11-month-old WT and IF rats. Scale bar = 50 μm.

(C) The number of eMHC-positive fibers per TA section of 1, 6, 11-month-old WT and IF rats. Data are presented as means+SD (n=3-4, in each group). NP= not present.

(D) The number of Pax7(+) satellite cells per well on day 2 in primary culture from 6, 11-month-old WT and IF rats. Bars represent the mean value of each group. *$P < 0.05$, by t-test.

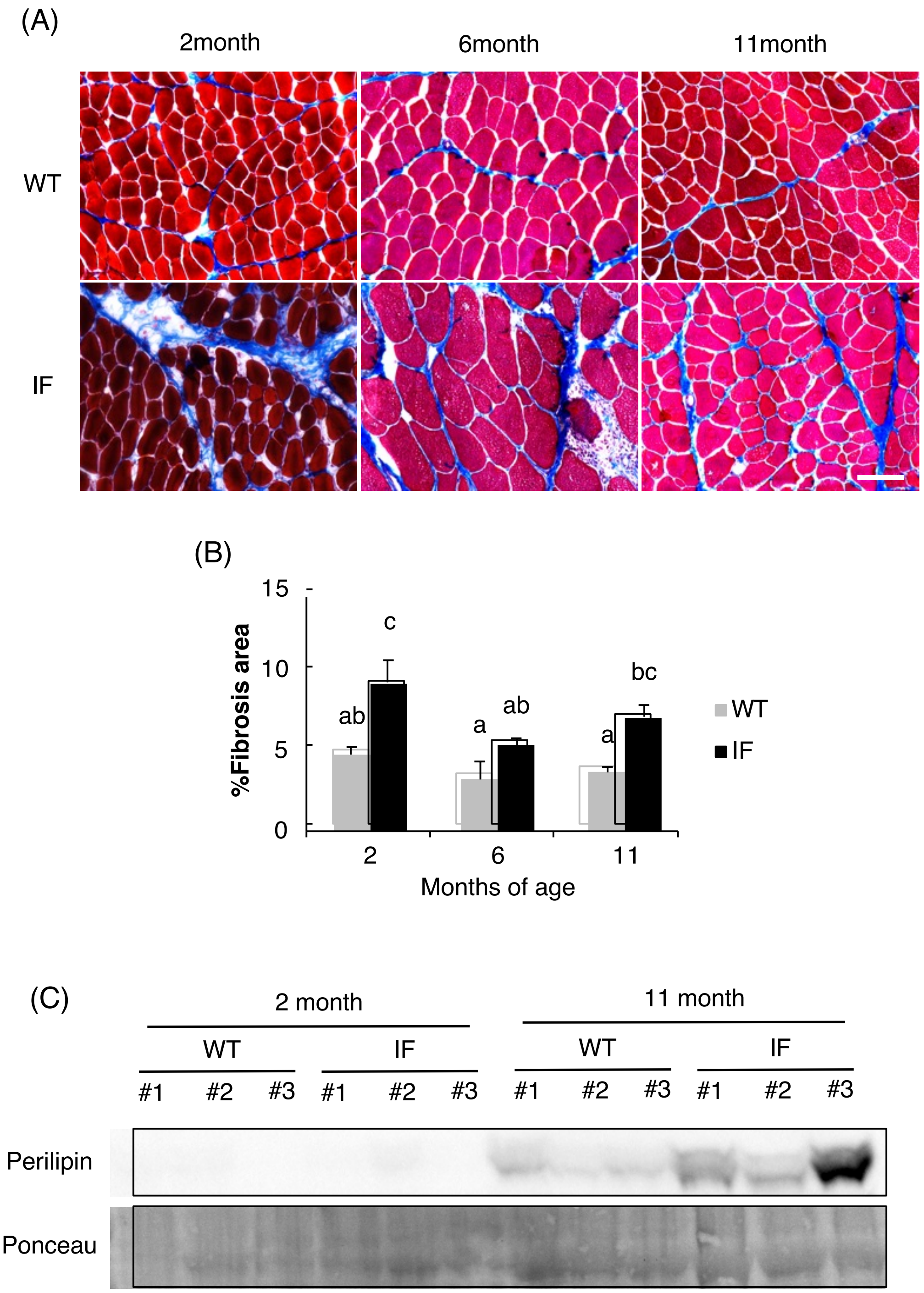

Figure. S2 Promoted fibrosis and accumulation of adipose tissues in skeletal muscles of IF rats. (A) Representative images of Masson trichrome staining on TA muscle sections of 2, 6, 11-month-old WT and IF rats. Scale bar = 50 μm.

(B) Percentage of fibrosis area in total area observed in TA muscle sections of 2, 6, 11-month-old WT and IF rats. Different letters indicate significant differences between groups (P<0.05, by Tukey's test). Data are presented as means+SD (n=3, in each group).

(C) Immunoblot analysis of perilipin protein expression in TA of 2, 11-month-old WT and IF rats. Ponceau S staining was used as a loading control.

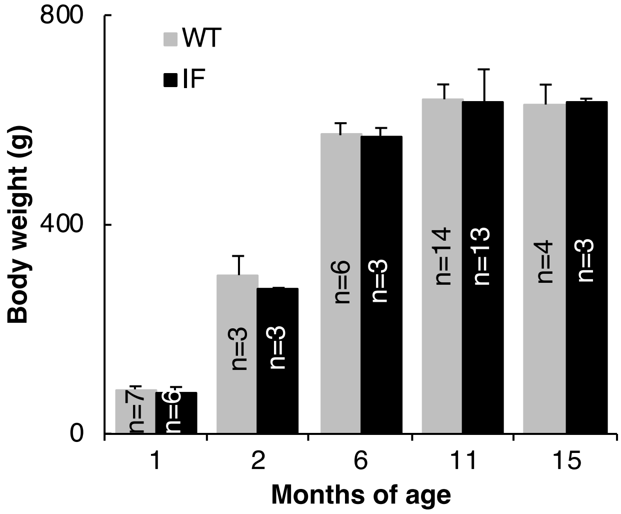

Figure. S3 Body weight of WT and IF rats
Body weight of 1, 2, 6, 11, 15-month old WT and IF rats. Data are presented as means+SD (n=3-14, in each group).

Disease Models & Mechanisms • Supplementary information

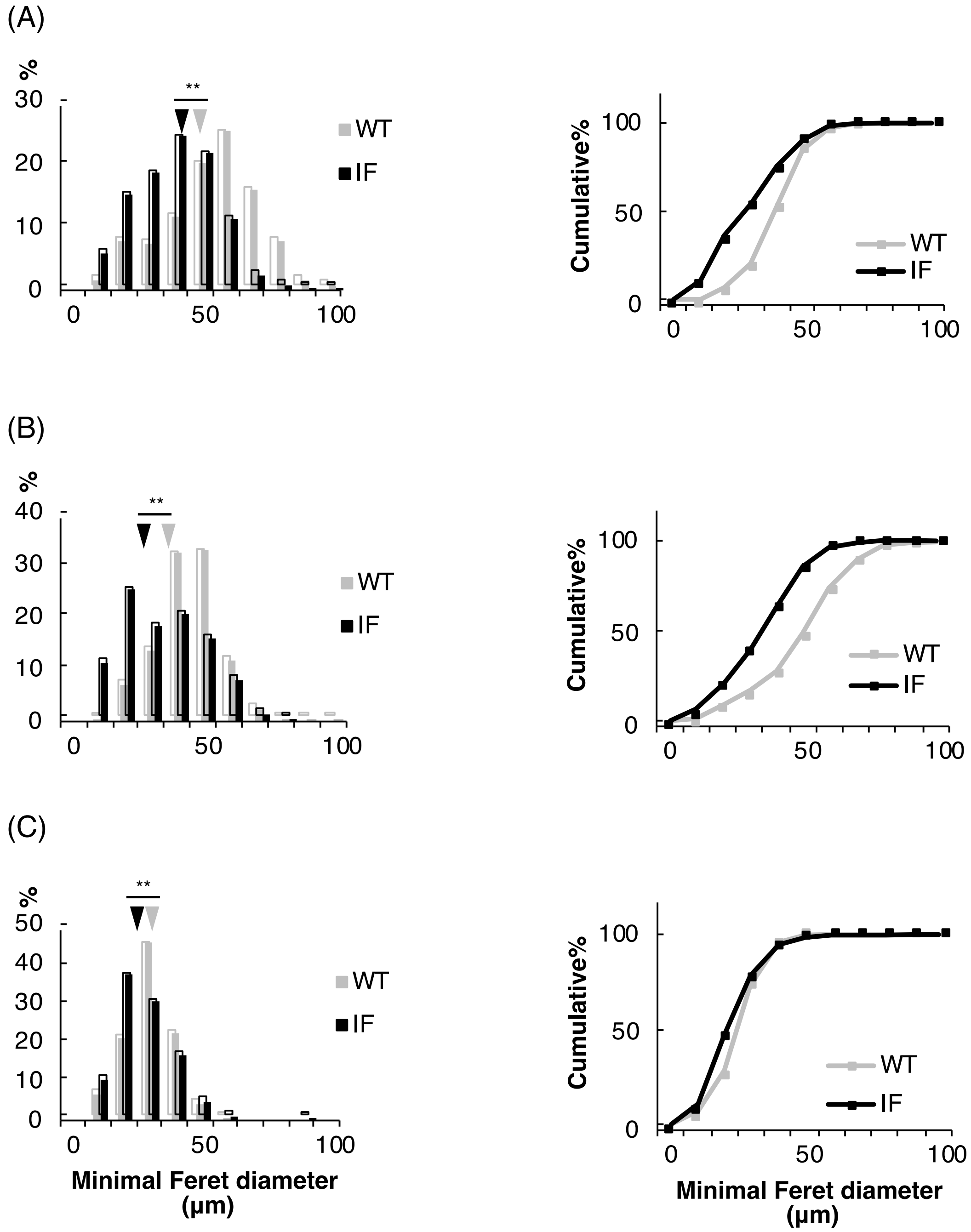

Figure. S4 IF soleus muscles have reduced myofiber size compared with WT.
Relative distributions and cumulative plots of myofiber size in SOL at 2- (A), 6- (B), and 11-month-old (C) WT and IF rats. Each arrowhead indicates a median value of each group. The data in each group contains the total number of detected myofibers from 3 subjects per group. $^{**}P < 0.01$, analysis using Wilcoxon rank sum test.

Disease Models & Mechanisms • Supplementary information

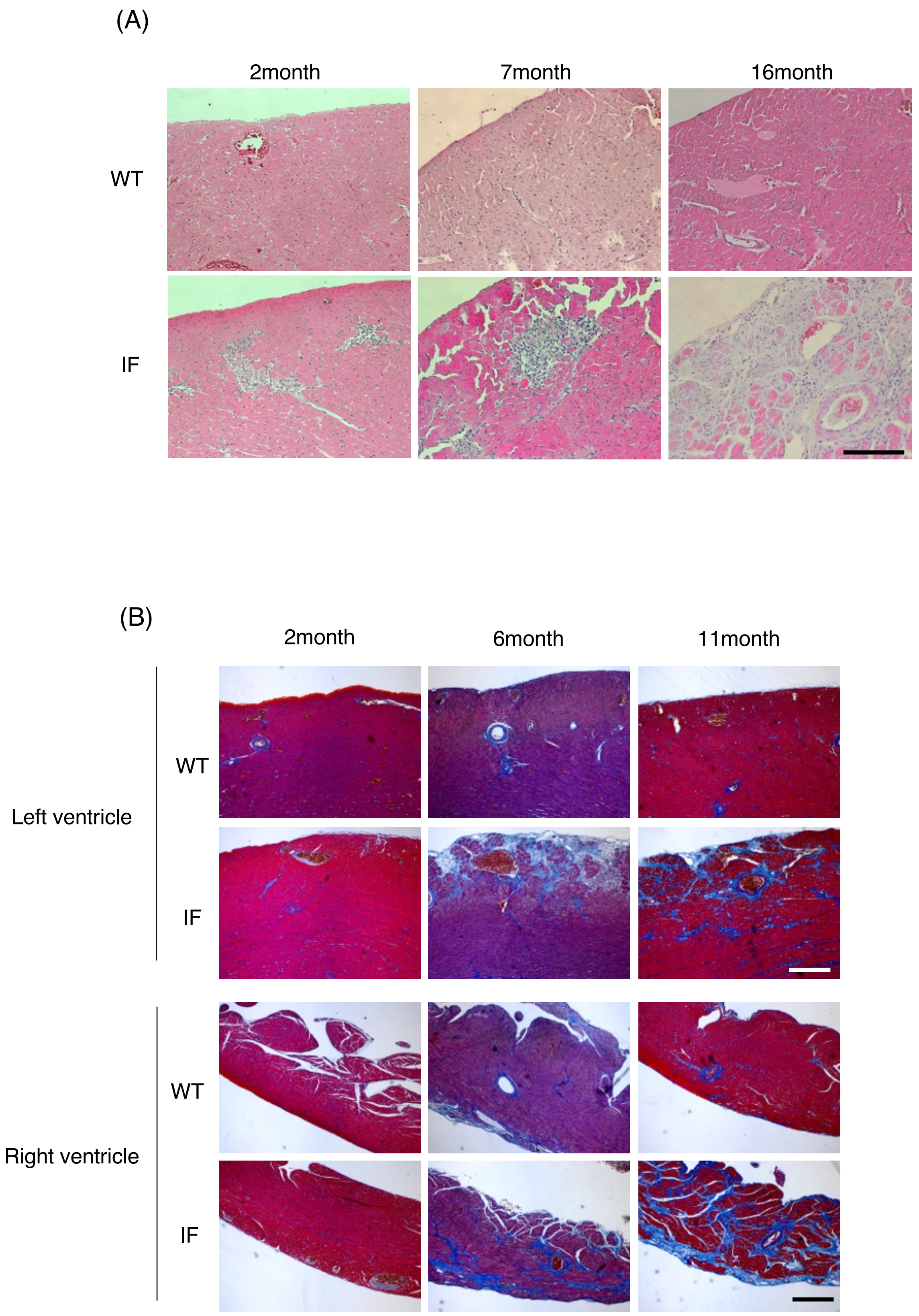

Figure. S5 Continuous degeneration and progression of fibrosis in cardiac muscles of IF rats.
(A) Representative images of H&E staining on left-ventricular-wall sections of 2, 7, 16-month-old WT and IF rats. Scale bar = 100 μm.
(B) Representative images of Masson trichrome staining on left- and right-ventricular-wall sections of 2, 6, 11-month-old WT and IF rats. Scale bar = 100 μm.

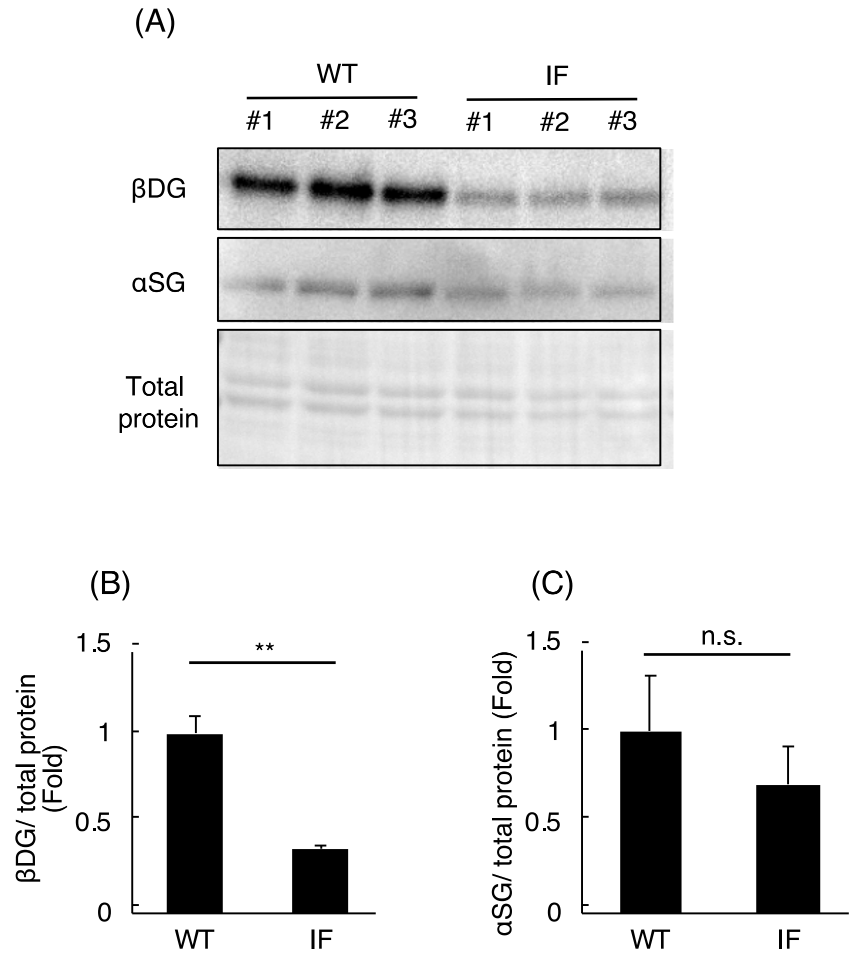

Figure. S6 Reduction of DGC components expression in skeletal muscle of IF rats.
(A) Immunoblot analysis of β-dystroglycan and α-sarcoglycan protein expression in TA of 1-month-old WT and IF rats. Ponceau S staining was used as loading control.
(B, C) β-dystroglycan (B) and α-sarcoglycan (C) expression levels quantified by the immunoblot analysis shown in figure S5A. Data are presented as means+SD (n=3). $^{**}P < 0.01$, n.s.=not significant, by t-test.

Disease Models & Mechanisms • Supplementary information

Table S1. Other Echocardiographic Variables

Variables	7 month			16 month		
	WT (4)	IF (4)	P (t-test)	WT (5)	IF (5)	P (t-test)
Heart rate (bpm)	327 ± 18	354 ± 29	0.221	335 ± 19	332 ± 29	0.869
**Left Heart Variables**						
Septal wall thickness (mm)	1.7 ± 0.3	1.6 ± 0.4	0.769	1.7 ± 0.1	1.5 ± 0.3	0.315
Posterior wall thickness (mm)	1.9 ± 0.1	1.8 ± 0.1	0.363	1.8 ± 0.3	1.8 ± 0.2	0.774
LV end-diastolic diameter (mm)	9.5 ± 0.6	8.7 ± 0.4	0.12	10.1 ± 0.6	9.8 ± 0.4	0.492
LV end-systolic diameter (mm)	6.2 ± 0.7	5.1 ± 0.1	0.037	6.1 ± 1.3	5.8 ± 1.0	0.727
LV fractional shortening (%)	35.4 ± 3.8	42.0 ± 3.5	0.069	39.5 ± 11.7	40.7 ± 10.2	0.888
Transmitral E velocity (cm/s)	110 ± 23	126 ± 7	0.281	118 ± 19	104 ± 18	0.344
Transmitral E deceleration time (ms)	53 ± 16	42 ± 4	0.302	39 ± 8	49 ± 11	0.164
LV myocardial performance index	0.33 ± 0.05	0.26 ± 0.06	0.194	0.27 ± 0.04	0.33 ± 0.13	0.351
Sm septum (cm/s)	3.8 ± 0.7	4.2 ± 1.1	0.685	3.4 ± 0.3	4.0 ± 0.5	0.085
Sm lateral (cm/s)	6.1 ± 0.9	5.2 ± 1.0	0.296	5.9 ± 0.7	4.5 ± 0.7	0.021
Ea septum (cm/s)	6.9 ± 2.8	5.9 ± 2.0	0.640	6.4 ± 0.5	5.0 ± 1.5	0.149
Ea lateral (cm/s)	6.3 ± 1.7	6.1 ± 2.3	0.912	5.7 ± 1.5	4.7 ± 0.9	0.305
Transmitral E/Ea septum	18.8 ± 8.2	23.5 ± 6.5	0.465	18.6 ± 4.6	22.2 ± 5.5	0.383
Transmitral E/Ea lateral	18.4 ± 4.5	22.7 ± 5.7	0.342	22.2 ± 6.3	23.1 ± 5.9	0.833
**Right Heart Variables**						
RV fractional area change (%)	41.5 ± 3.7	44.0 ± 3.8	0.457	41.3 ± 6.1	39.5 ± 6.5	0.683
TAPSE (mm)	1.8 ± 0.5	2.3 ± 0.3	0.189	2.3 ± 0.3	1.5 ± 0.6	0.061
Transtricuspid E velocity (cm/s)	67.2 ± 19.8	77.1 ± 12.3	0.191	84.7 ± 5.4	72.2 ± 18	0.666
Sm RV free-wall (cm/s)	5.6 ± 0.4	5.1 ± 0.3	0.096	4.6 ± 0.7	3.9 ± 0.9	0.279
Ea RV free-wall (cm/s)	7.9 ± 2.2	5.5 ± 1.5	0.425	9.1 ± 0.7	6.1 ± 3.4	0.357
Transtricuspid E/Ea RV free-wall	9.0 ± 2.8	15 ± 4.9	0.842	9.4 ± 1.1	12.3 ± 6.4	0.525
Maximum IVC diameter (mm)	3.6 ± 1.0	2.6 ± 0.4	0.161	3.1 ± 0.8	3.0 ± 0.3	0.849

Table S1 Values from echocardiography
*P<0.05, WT vs age-matched IF, by t-test. Numbers in parentheses represent the number of subject in each group.

Table S2 List of primers

		Forward	Reverse
Fig. 1C	genome #1	5'-AAAAGGAGAACAGGAGTTTTTGAAT-3'	5'-TACAGTAGCTGAGTCAATGAGGTTG-3'
	genome #2	5'-GAATACCTTTGGGTGTGACTGTATC-3'	5'-TACAGTTTTCCATTTCTGAAGAACC-3'
Fig. 1D	mRNA #1	5’-AAAGCAACACATAGACAACCTCTTC-3'	5'-CCTCTTGGGCATGTTTTACCA-3'
	mRNA #2	5'-GAACTCAGCTCTTGAAGGCAAT-3'	5'-CTTCCAAAGTTTTGCATTTTCC-3'
Fig. 4D	*Dmd*	5'-GGAAGATCTGAATACCAGATGGA-3'	5'-CTGCCTGACACGGTCCTC-3'
	Hprt	5'-GACCGGTTCTGTCATGTCG-3'	5'-ACCTGGTTCATCATCACTAATCAC-3'
